# The Relevance of Plant-Based Diets and Micronutrient Supplementation for Body Composition: Data from the VeggieNutri Cross-Sectional Study

**DOI:** 10.3390/nu16193322

**Published:** 2024-09-30

**Authors:** Cátia Pinheiro, Flávia Silva, Inês Rocha, Carina Martins, Liliana Giesteira, Bruna Dias, Ana Lucas, Ana Margarida Alexandre, Catarina Ferreira, Bruna Viegas, Isabella Bracchi, Juliana Guimarães, Joana Amaro, Teresa F. Amaral, Cláudia Camila Dias, Andreia Oliveira, Altin Ndrio, João Tiago Guimarães, João Costa Leite, Rita Negrão, Elisa Keating

**Affiliations:** 1 Unit of Biochemistry, Department of Biomedicine, Faculty of Medicine, University of Porto, 4200-319 Porto, Portugalritabsn@med.up.pt (R.N.); 2 CINTESIS, Center for Health Technology and Services Research, 4200-319 Porto, Portugal; 3 Faculty of Health Sciences, Fernando Pessoa University, 4249-004 Porto, Portugal; flaviaafsilvaa@gmail.com (F.S.);; 4 Faculty of Nutrition and Food Sciences, University of Porto, 4150-180 Porto, Portugalviegas.b@hotmail.com (B.V.); tamaral@fcna.up.pt (T.F.A.); 5 EPIUnit–Institute of Public Health, University of Porto, 4050-600 Porto, Portugal; jmcamaro@gmail.com (J.A.);; 6 Laboratory for Integrative and Translational Research in Population Health (ITR), University of Porto, 4050-600 Porto, Portugal; 7 Department of Medicine, Faculty of Medicine, University of Porto, 4200-319 Porto, Portugal; 8 INEGI—Institute of Science and Innovation in Mechanical and Industrial Engineering, LAETA—Associate Laboratory for Energy, Transports and Aerospace, 4200-465 Porto, Portugal; 9 CINTESIS@RISE, Department of Community Medicine, Information and Health Decision Sciences (MEDCIDS), Faculty of Medicine, University of Porto, 4200-319 Porto, Portugal; 10 Department of Public Health and Forensic Sciences and Medical Education, Faculty of Medicine, University of Porto, 4200-319 Porto, Portugal; 11 Clinical Pathology, São João University Hospital Center, 4200-319 Porto, Portugal; 12 Joint Research Centre, European Commission, 21027 Ispra, Italy; 13 CINTESIS@RISE, Faculty of Medicine, University of Porto, 4200-319 Porto, Portugal

**Keywords:** anthropometry, body composition, nutritional status, vegan diet, vegetarian diet, lacto-ovo-vegetarian diet, omnivorous diet, plant-based diets, micronutrient supplements, muscle mass

## Abstract

Objectives: This study aimed to compare the anthropometry and body composition of vegetarian and omnivorous adults living in Portugal, while exploring nutritional and health parameters underlying observed differences. Methods: 425 omnivorous (OMNI), lacto-ovo-vegetarian (LOV), or vegan (VEG) healthy adults were recruited. Anthropometry was measured, and bioelectrical impedance analysis was performed. Participants answered food frequency, sociodemographic, and lifestyle questionnaires. Serum iron, ferritin, and CRP were quantified by spectrophotometry, and serum B12 vitamin and homocysteine were quantified by chemiluminescent microparticle immunoassay. Results: Total protein intake significantly decreased with increasing strictness of vegetarian habits (median (P25; P75) in g/day: 98.6 (79.5; 123.1), 90.4 (65.9; 121.0), and 87.6 (59.8; 118.5) for OMNI, LOV and VEG, respectively; *p* = 0.020), and carbohydrate intake was the highest in LOV (median (P25; P75) in g/day: 231.5 (178.4; 287.9), 283.9 (227.3; 342.6), and 263.0 (222.0; 348.3) for OMNI, LOV and VEG, respectively; *p* = 0.001). VEG were the main users of B12 vitamin (93% in VEG vs. 17% in OMNI and 59% in LOV, *p* = 0.001), and LOV were the main users of iron supplements (29% in LOV vs. 14% in OMNI and 13% in VEG, *p* = 0.042), respectively. Blood levels of B12 vitamin correlated negatively with blood homocysteine (*r_s_* = −0.386, *p* < 0.001) and positively with % muscle mass (*r_s_* = 0.136, *p* = 0.005). Participants using iron supplements presented higher C-reactive protein (CRP) (*p* = 0.014) and they had lower % MM (*p* = 0.003). Finally, when compared to being OMNI, being LOV independently associated with: (a) having +4.8% (*p* = 0.002) of fat mass, which could be due to higher carbohydrate intake, and (b) having −2.2% (*p* = 0.043) of muscle mass. Our data suggest that the association between diet and muscle mass could be attenuated in VEG due to B12 supplementation and/or aggravated in LOV due to iron supplementation-associated inflammation.

## 1. Introduction

A dietary shift towards plant-based diets has been linked with positive environmental and health outcomes [1,2,3], highlighting the importance of reducing the consumption of animal-based foods. The latest Portuguese National Food, Nutrition, and Physical Activity Survey (IAN-AF) [4] has found that current diets are not in line with the national dietary guidelines. In fact, the Portuguese population is consuming a higher amount of foods from the “meat, fish and eggs” and “dairy products” groups (+12% and +6%, respectively) and a lower amount of foods from the groups of “vegetables”, “fruit” and “legumes” (−8%, −7% and −2%, respectively) [4], than the current recommendations of the Portuguese food-based dietary guidelines [5]. Additionally, results from the same survey show that Portuguese adults are consuming on average 126.7 g of meat per day [4], which corresponds to approximately three times the meat portion recommended by the latest scientific targets for planetary health by the EAT-Lancet Commission, of about 43 g/day [6].

Reducing consumption of animal-source foods and, in particular, ruminant meat, is central to improving the sustainability of the food system and to contributing to climate commitments of the country. However, a better understanding of the potential nutritional and health value of a transition towards plant-based diets in the national context is needed to inform people about the opportunities and risks for better dietary guidance.

Vegetarian diets are good examples of plant-based diets, and they include: vegan or strictly vegetarian diets (VEG), which completely exclude all animal-derived foods; lacto-ovo-vegetarian diets (LOV), which exclude meat and fish but include eggs and milk/dairy; ovo-vegetarian diets, which exclude meat, fish, and milk/dairy but include eggs; and lacto-vegetarian diets, which exclude meat, fish, and eggs and include milk and dairy products. All these diets have a significantly lower environmental footprint than omnivorous (OMNI) dietary patterns [7,8,9].

Knowledge on the proportion of the population following vegetarian diets is scarce in several countries, yet the overall proportion in some Western countries is estimated to vary between 1% and 10% [10]. Still, there are no official reports on the prevalence of vegetarianism in Portugal, and the only available data is from a market research from 2021, which estimated that the prevalence of Portuguese following a vegetarian dietary pattern was 2.6% [11].

Literature has shown several health benefits of vegetarian diets [12,13,14,15,16]. Several studies report that participants allocated to vegetarian diets lose more weight and present a lower BMI with [12] and without [12,13,14] energy restriction, compared to those allocated to omnivorous diets. These reductions have been shown to be more pronounced in vegans, followed by lacto-ovo-vegetarians [14]. Additionally, vegetarian diets have been associated with fat mass reduction (either total fat mass or visceral fat) [12], lower concentrations of total LDL and HDL cholesterol [15], as well as reduced risk of diabetes, several cancers [15,16], isolated diastolic hypertension [16] or ischemic heart disease [15].

The health-beneficial effects of vegetarian diets are often attributed to a low intake of saturated fats and, on the other hand, to a high intake of a vast range of fibers, sterols, and phytochemicals that are abundant in plant-based foods [16,17].

Nevertheless, vegetarian diets may be associated with nutritional inadequacies such as excessive intake of highly processed and refined foods or deficiency in B12 vitamin and iron [17,18,19,20,21]. In human metabolism, iron and B12 vitamin have crucial roles: iron is recruited for erythropoiesis and hormone production and it is a cofactor for enzymes involved in oxidative phosphorylation, and B12 vitamin is used as a coenzyme in the production of red blood cells, DNA and it is also recruited in the acid-folic metabolism [22]. On the one hand, iron from plant foods occurs in the non-heme form, and it is less bioavailable than the heme iron present in animal-sourced foods [22]. On the other hand, B12 vitamin is only ingested in substantial quantities through the consumption of animal-sourced foods, thus making some vegetarians and vegans dependent on the consumption of fortified foods, food additives, or nutritional supplements to ensure their B12 daily needs [22,23].

In accordance, although vegetarian diets, when well planned, are considered healthful and nutritionally adequate [7,24,25], they may give rise to nutritional inadequacies, such as poor micronutrient status, if they are adopted without nutritional counselling [26].

In this context, the putative rise of vegetarian habits among Portuguese population [11] and the current overall lack of information on this issue, namely vegetarians’ sociodemography, lifestyle, anthropometry, body composition, and aspects such as nutritional counselling and supplement intake, pose important public health concerns. In fact, this characterization is urgently needed to empower healthcare professionals and public health entities to act on the best practices of dietary guidance for these specific population groups in Portugal.

Therefore, the present work aims to characterize the anthropometry and body composition of vegetarian (VEG and LOV) adults living in Portugal, comparing these parameters with the ones from a sample of OMNI individuals. In addition, this work explores how the intake of protein, fat, carbohydrate, iron, B12 vitamin, or inflammatory state could explain the observed differences.

## 2. Materials and Methods

### 2.1. Study Design and Participants

VeggieNutri (ClinicalTrials.gov #NCT05408962) was performed according to the protocol approved by the Ethics Committee of the Faculty of Medicine of the University of Porto (#23/CEFMUP/21) and validated by the Data Protection Office of the University of Porto (#A-24/2021). Written informed consent was obtained from all participants.

VeggieNutri is a cross-sectional study that was conducted at the Faculty of Medicine of the University of Porto, Portugal. From December 2020 to February 2022, healthy Portuguese-speaking adults (aged 18 to 64 years old) living in Portugal were invited to participate through Portuguese mass media and mailing lists of different universities. In addition, collaborations with national associations related to vegetarianism were established to advertise the study through their online platforms. Healthy adults who had an OMNI, LOV or VEG dietary pattern for at least 1 year and who provided their consent to participate in the study were included. Exclusion criteria were: having any chronic diseases such as metabolic, digestive, renal, cardiovascular, hematological, endocrine, or oncological diseases (except for obesity and hypertension due to the very high prevalence of these two conditions in the Portuguese population [27,28,29]), having food allergies and intolerances or eating disorders, suspecting pregnancy or being pregnant and having a pacemaker or internal metallic prosthetics.

**Figure 1:**
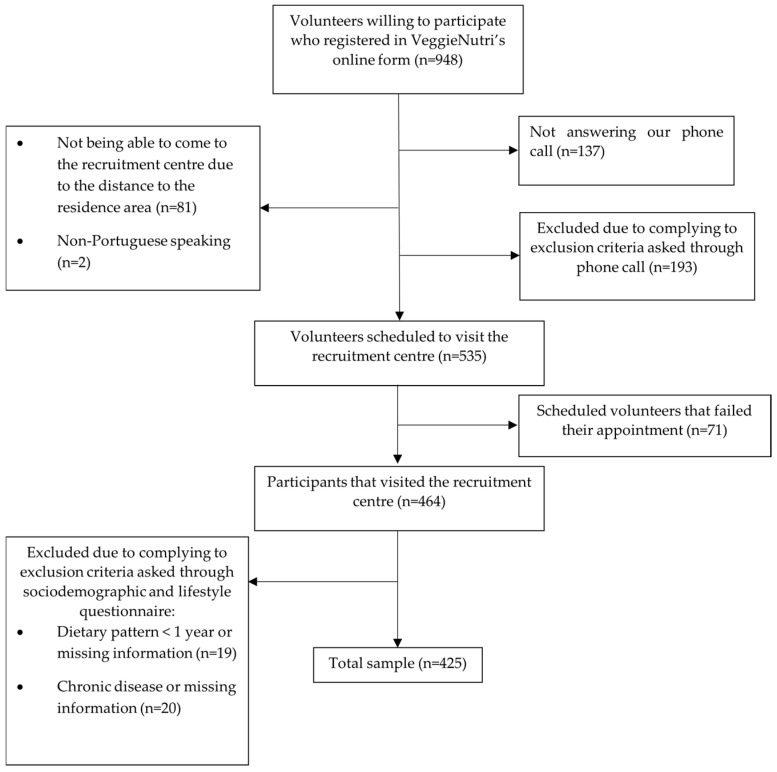
Recruitment flowchart of the VeggieNutri project.

On the day of the visit, blood and spot urine collection from 8 h fasting participants took place. In addition, participants answered a sociodemographic and lifestyle (dietary pattern and respective duration, supplementation intake, medication use, nutritional counseling, physical activity practice, and clinical history) questionnaire and a food frequency questionnaire, as described below. Finally, weight, height, waist circumference (WC), hand grip strength (HGS), and blood pressure were measured by trained researchers, and body composition was assessed (details on anthropometry, HGS, blood pressure, and body composition measurements are presented below). Figure 1 shows VeggieNutri’s recruitment flowchart.

### 2.2. Data Collection

#### 2.2.1. Food and Nutritional Intake Assessment and Diet Group Definition

To assess food and nutritional intake, a Food Frequency Questionnaire (FFQ) with adaptations regarding vegetarian dietary habits was applied to all participants. The FFQ is a retrospective method of food intake assessment that estimates the overall individual food intake regarding the previous 12 months, in which the participants register the frequency of their consumption within 9 categories ranging from “never or less than once a month” to “6 or more times a day” of 86 food items or food groups. To this original version of the FFQ, which is validated for the Portuguese adult population [30,31], 17 groups of foods and food items specifically consumed by vegetarians were added, according to unpublished data from the latest IAN-AF. The adapted FFQ was later used to estimate each participant’s overall daily consumption of energy, macronutrients and micronutrient, by using food composition tables, which have been adapted to traditional Portuguese foods. For the specific aims of this study, we obtained the overall consumption of the daily total protein intake in grams and the total daily intake of cobalamin (B12 vitamin) and iron in micrograms. Protein intake adequacy was classified into categories based on the IOM *Recommended Dietary Allowances and Adequate Intakes, Total Water, and Macronutrients* [32]. The following protein adequacy categories were considered: “below adequacy”, when protein intake was lower than 10% of the total energy intake; “within adequacy”, when protein intake was between 10 and 35% of the total energy intake; or “above adequacy”, when protein intake was higher than 35% of the total energy intake.

As for the definition of the dietary pattern, self-reported dietary patterns were first considered. This variable was defined as “self-reported diet”. Since some self-reported LOV and VEG participants indicated consumptions higher than “never or less than once a month” of the FFQ’s “meat, fish and eggs” and “milk/dairy” food groups, these participants were then re-categorized into the new category of corrected dietary pattern. This variable was defined as “real diet”, and it was used for the whole statistical analysis in this study.

Additionally, the self-reported intake (yes/no) of multivitamin, isolated B12 vitamin, or isolated iron supplements was obtained from the sociodemographic and lifestyle questionnaire.

#### 2.2.2. Anthropometric and Body Composition Assessment

All anthropometric and body composition measurements were conducted by a trained nutritionist.

Weight and height were assessed according to the International Standards for Anthropometric Assessment Protocol standardized by the International Society for the Advancement of Kinanthropometry, ISAK [33].

Weight was measured using a digital measuring scale (TANITA^®^ RD-953, TANITA CORP of America), according to ISAK methodology [33] with the participant standing in minimal clothing. With the scale reading zero, the participant stood on the center of the scale without support, with the arms pending next to the body, the head looking ahead, and with body weight evenly distributed by both feet. The measurement was considered when the value on the scale stabilized.

Height was measured in triplicate with a SECA^®^ 213 portable stadiometer (SECA, Hamburg, Germany), according to ISAK methodology [33], with the participant standing, having the feet together, and the heels, buttocks, and upper part of the back touching the stadiometer. The head was placed in the Frankfort plane, with the back of the participant’s head touching the stadiometer whenever possible. The participant was instructed to take and hold a deep breath, and the height measurement was taken at the end of the deep inward breath by the trained nutritionist by placing the headboard of the stadiometer firmly down on the vertex of the participant’s head, crushing the hair as much as possible. The mean of the three height measurements was used for analysis.

Waist circumference was measured in triplicate at the umbilicus level, to the nearest 0.1 cm, using a non-stretchable measuring tape, as described by others [34,35]. The mean of the three WC measurements was used for analysis.

Body composition was assessed through bioelectrical impedance analysis (BIA) using TANITA^®^ RD-953 (TANITA CORP of America). BIA is often used in nutrition research for body composition assessment, being a non-invasive and simple exam to perform [36]. Data on fat mass (FM), muscle mass (MM), bone mass (BM), visceral fat (VF), and total body water (TBW) were obtained. BMI was calculated by the TANITA^®^ during body composition assessment, and the WHO cut-off points for adults were used for BMI classification [37].

Body composition assessment was run once, with the participant wearing minimal clothing. The instructions for the measurements were the same as previously mentioned in the weight assessment. To be submitted to BIA, participants complied with the following requirements: being in a fasting condition for at least 8 hours; having avoided alcoholic beverages and caffeine-rich foods consumption the day before the BIA exam; having avoided performing intense physical exercise (of more than 45 min of vigorous physical exercise) the day before the exam; as well as having avoided using the sauna or Turkish bath the day before. Also, participants had to avoid taking the exam while menstruating and had to remove all metallic objects (excluding orthodontic braces or dental implants) before performing the exam.

In addition, muscle mass (in kg) obtained by BIA was divided by body weight (in kg) and multiplied by 100 to obtain the percentage of muscle mass adjusted to total body weight (% MM).

#### 2.2.3. Blood Pressure, Heart Rate, and HGS Measurements

Blood pressure was measured according to the Clinical Practice Guidelines for the Management of Hypertension in the Community [38] using an electronic sphygmomanometer (OMROM 3 Series^®^ Upper Arm Blood Pressure Monitor, OMRON Corporation, Kyoto, Japan) on the brachial artery by a research nurse. Participants were comfortably seated in a chair with their legs resting on the ground in an uncrossed position. Two measurements of systolic and diastolic blood pressure were taken, with a 1–2-minute interval between measurements, and the average of these measurements was calculated.

HGS was measured with a Jamar^®^ Smart Digital Hand Dynamometer (Sammons Preston Inc., Bolingbrook, IL, USA). Three consecutive measurements were taken using the non-dominant hand, which was self-reported by participants, and the mean value was used for analysis. HGS measurements were conducted accordingly to the American Society of Hand Therapists [39]: participants seated comfortably with their shoulder abducted and neutrally rotated and the elbow of the non-dominant arm folded as close to 90 degrees as possible. The forearm was in a neutral position with the wrist between 0 and 30 degrees dorsiflexion and between 0 and 15 degrees ulnar deviation. Participants gripped the dynamometer with their fingers around the handle, with the readout dial pointing away from their body. Then, on a three-to-zero countdown, participants were instructed to squeeze the dynamometer handle with their maximum force for 3 seconds, followed by a 15 second interval before the following measurement.

#### 2.2.4. Iron, Ferritin, B12, CRP, and Homocysteine Blood Analysis

8 h fasting blood samples were collected. Analytes were quantified according to the respective manufacturer’s instructions. Serum iron, ferritin, and CRP were quantified by spectrophotometry in an AU5800 clinical chemistry analyzer (Beckman Coulter, Inc., Brea, CA, USA) and serum B12 vitamin and homocysteine were quantified by chemiluminescent microparticle immunoassay in an ARCHITECT immunoassay analyzer (Abbott Core Laboratory, IL, USA). According to manufacturers, intra- and inter-assay coefficients of variation, in %, were between 0.5 and 1.1, respectively, for iron; between 0.61 and 2.24, respectively, for ferritin; and between 2.5 and 6.9, respectively, for B12 vitamin.

Sex-specific reference levels considered for each analyte were set by the Clinical Pathology Department of CHUSJoão, Porto, Portugal.

### 2.3. Statistical Analysis

Sociodemographic, lifestyle, anthropometric and body composition data from all three groups were analysed using the IBM^®^ SPSS^®^ Statistics version 26 software and statistical significance was set to a *p* value < 0.05.

The sample size was defined as optimal based on adequate procedures to detect significant differences between the dietary food groups with 80% power and a 95% confidence level for primary outcomes.

Descriptive statistics for categorical variables are presented as absolute (n) and relative (%) frequencies, and continuous variables were described as mean and standard deviation (mean ± SD) or medians and the 25th and 75th percentiles (P25; P75) depending on the skewness of the distribution. There was no missing data regarding eligibility variables, including “real diet” and food intake assessment. Missing data were as follows: for anthropometry, between 0% and 0.2% (n = 0–1) of the total sample; for body composition, between 0 and 0.5% (n = 0–2) of the total sample; and for blood biomarkers, 0.2% (n = 1) of the total sample.

For the hypothesis tests, the Chi-square or exact Fisher test was used for categorical variables as appropriate; one-way ANOVA was used for the continuous and normally distributed variables (such as age, height, both systolic and diastolic blood pressure, and muscle mass adjusted for weight), and the Kruskal–Wallis test was applied to test for differences in the median of continuous but not normally distributed variables such as residence area, marital status, educational level, weight, BMI, HGS, WC, FM, MM, BM, VF, and TBW, according to the dietary pattern (VEG, LOV, or OMNI).

To test if the statistically significant associations found were attributed to the dietary pattern (independent variable), multiple linear regression models were run. Based on previous literature and exploratory statistics, the following variables were used as potential confounding variables: sex, age, marital status, self-reported multivitamin, isolated vitamin B12 and isolated iron supplement intake, and nutritional counselling (yes/no).

Furthermore, Spearman correlations were conducted to test for the association between blood levels of B12 vitamin and % muscle mass and blood levels of B12 vitamin and blood level of homocysteine.

Subgroup analyses were conducted based on the duration of diet adherence to assess its impact on body composition, anthropometric, and biochemical parameters. According to literature [40,41], we assumed 5 years as a cut-off point for the duration of diet adherence subgroup analyses.

## 3. Results

### 3.1. Sociodemographic Characterization

The total sample included 425 individuals. Distribution by self-reported diets was 58% (n = 247) OMNI, 25% (n = 106) LOV, and 17% (n = 72) VEG. By using data from the FFQ on the consumption of animal-source foods, individuals were re-allocated in real diet groups as 62% (n = 263) OMNI, 23% (n = 98) LOV, and 15% (n = 64) VEG, indicating that 8% of our total sample incorrectly self-identified their dietary pattern (Supplementary Materials Table S1).

Participants adhered for a minimum of 1 and a maximum of 25 years to the LOV diet and for a minimum of 1 and a maximum of 24 years to the VEG diet.

Table 1 presents the sociodemographic and lifestyle characteristics of the total sample and stratified by real diet groups.

Global mean (SD) age was 36 (12) years, with LOV and VEG being younger than OMNI, *p* = 0.001.

The majority of participants were female both in the total sample and in the different dietary groups. The great majority of the total sample were of Portuguese nationality (90%), lived in the North of Portugal (91%), and had a high educational level (77%), defined as 13 or more completed years of school. Regarding marital status, most of our sample was composed of single individuals, with the highest proportion of single individuals in the LOV group (*p* = 0.028). Additionally, for the household monthly income, most of our sample (65%) earned two or more minimum wages (>1410.00€).

Regarding lifestyle characteristics, the majority of our participants were non-smokers (78%) and practiced physical exercise (63%), and, among these, the majority (63%) practiced at least 3 times a week. These characteristics were not different between dietary patterns.

The proportion of participants taking nutritional supplements was the highest in the VEG (*p* = 0.001) group. Regarding the type of supplements, OMNI were the main users of multivitamins (*p* = 0.001), VEG were the main users of vitamin B12 (*p* = 0.001), and LOV were the main users of iron supplements (*p* = 0.042).

The proportion of participants seeking nutritional counselling was generally low, but it was higher in vegetarian groups (*p* = 0.001). Among the participants using nutritional counselling, the highest proportion sought counselling from a nutritionist.

### 3.2. Anthropometry, Body Composition, and Health Characteristics by Dietary Pattern

Table 2 exhibits the anthropometric and body composition of the study sample.

Weight, height, and BMI were similar among dietary patterns. Median BMI was within adequacy in all groups, and 33% of the sample was overweight or obese, with similar proportions in all diet groups. Accordingly, the majority of the sample was classified as having normal weight as defined by the WHO BMI classification [37], and this distribution did not differ between dietary patterns. WC was approximately 3 cm lower in LOV and VEG when compared to OMNI (*p* = 0.010). FM, bone mass, MM %, and HGS did not significantly differ between dietary patterns. Additionally, VF and TBW were adequate in all groups, but VF was the lowest and TBW was the highest in the VEG group (*p* = 0.033 for VF and *p* = 0.009 for TBW). Duration of the plant-based diet did not associate with any of the body composition, anthropometric, or biochemical parameters studied. There was an exception for visceral fat in the LOV group, which was higher for higher diet duration. This difference may be explained by age, since in LOV (but not in VEG) higher diet duration is associated with higher age (Supplementary Materials Tables S2 and S3).

Mean blood pressure (systolic and diastolic) was not significantly different among dietary patterns (mean systolic blood pressure ± SD, in mmHg, for OMNI, LOV, and VEG: 112 ± 15, 109 ± 12, and 110 ± 15, *p* = 0.304, respectively, and mean diastolic blood pressure ± SD, in mmHg, for OMNI, LOV, and VEG: 74 ± 10, 73 ± 8, and 73 ± 8, *p* = 0.406, respectively). The great majority of participants from all groups had systolic and diastolic blood pressure values below the cut-offs for the definition of hypertension [42], and these proportions were similar among dietary patterns (systolic blood pressure < 140 mmHg: 95% in OMNI, 98% in LOV, and 97% in VEG, and diastolic blood pressure < 90 mmHg: 95% in OMNI and 97% in LOV and VEG).

Multiple linear regression models were run to study the association between dietary patterns (independent variable–exposure) and body composition, anthropometry, blood pressure, and HGS (dependent variables–outcome), considering the following as potential confounding variables: sex, age, marital status, physical exercise, supplement intake (multivitamin, isolated B12 vitamin, and isolated iron), and nutritional counselling.

The crude models showed: (a) a negative association between WC or VF with LOV and VEG diet groups; (b) a positive association between FM and LOV diet groups; (c) a negative association between BMI and VEG diet groups; and (d) a positive association between TBW and VEG diet groups (Supplementary Materials Table S4). In this univariate analysis, no statistically significant associations were found between weight, height, bone mass, MM, % MM, HGS, or blood pressure (systolic and diastolic) and the diet groups.

The associations found between WC, VF, BMI, and TBW and dietary groups were lost after adjustment for sex, age, marital status, physical exercise, multivitamin supplement intake, isolated B12 vitamin supplement intake, isolated iron supplement intake, and nutritional counselling, meaning that dietary pattern does not independently explain these findings (Table 3).

On the other hand, after adjustment, being LOV was associated with having more 4.8% of fat mass and less 2.2% of muscle mass compared to being OMNI (Table 3). In VEG, % fat mass tended to be higher and % muscle mass tended to be lower when compared to OMNI, but these associations were not statistically significant (*p* = 0.400 and 0.589, respectively) (Table 3).

No associations were found between the dietary patterns and systolic or diastolic blood pressure on the crude models (Supplementary Materials Table S4) or when sex, age, marital status, physical exercise, multivitamin supplement intake, isolated B12 vitamin supplement intake, isolated iron supplement intake, and nutritional counselling were considered as covariates in the regression models (Table 3).

### 3.3. Food Intake and Blood Biomarkers in Relation to Body Composition

In order to explore the observed differences in % FM, % MM, and TBW between diet groups, the relevant nutrient intake and respective blood biomarkers were analysed.

**Figure 2:**
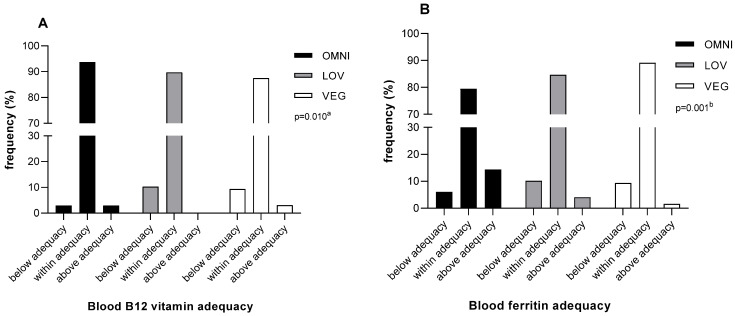
Sample distribution among adequacy categories for blood B12 vitamin (**A**) and ferritin (**B**), according to dietary group (LOV, lacto-ovo-vegetarians; OMNI, omnivorous; VEG, vegans). Sex-specific reference levels for blood ferritin and blood B12 vitamin set by the Clinical Pathology Department of CHUSJoão, Porto, Portugal were used as cut-offs of adequacy (Supplementary Materials Table S5). Data are presented as relative frequencies. ^a^ Fisher’s exact test; ^b^ Chi- square test.

Intakes of total carbohydrate, fat and energy, protein, iron, B12 vitamin, excluding dietary supplement intake, and water from foods were assessed. Moreover, blood iron, ferritin, and B12 vitamins were assessed for each diet group. Results are presented in Table 4 and in Figure 2.

Total water ingestion through foods was not different amongst the three dietary patterns.

Total carbohydrate intake from food consumption differed between groups, being the highest in the LOV group (*p* < 0.001). Similarly, total fat intake and total energy intake tended to be higher in LOV when compared with the other dietary patterns, but this difference was not statistically significant.

Total protein and B12 vitamin intake from foods significantly decreased with increasing strictness of vegetarian habits (*p* = 0.020, *p* = 0.001, and *p* = 0.001, for OMNI, LOV, and VEG, respectively). Additionally, the prevalence of below adequate protein intake also increased with increasing strictness of vegetarian habits (Table 4). On the other hand, blood levels of B12 vitamin were higher in the VEG group (*p* = 0.001) (Table 4), most likely due to a higher prevalence of B12 supplementation in this group (Table 1). Despite this supplementation, when compared to OMNI, the proportion of VEG individuals below adequacy for B12 vitamin blood levels was higher (9.4% for VEG vs. 3% for OMNI), and the proportion of VEG individuals within adequacy was lower (87.5% for VEG vs. 93.7% for OMNI) (*p* = 0.010) (Figure 2A).

Both LOV and VEG groups presented a higher dietary iron intake than the OMNI group (*p* = 0.001), yet blood iron levels were not different between groups, and blood ferritin decreased with increasing strictness of vegetarian habits (*p* = 0.001) (Table 4). In agreement with this, the prevalence of participants with blood ferritin values below adequacy was higher for vegetarian participants when compared to OMNI (10.2% for LOV and 9.4% for VEG vs. 6.1% for OMNI) (*p* = 0.001) (Figure 2B). This decrease in iron status did not seem to be affected by iron supplementation, which was more prevalent in the LOV group (Table 1). In fact, iron supplementation did not associate with an increase in blood iron or in ferritin levels (Table 5).

### 3.4. B12 and Iron Supplementation in Association with Muscle Mass

Considering that in our sample (a) being LOV associated with a 2.2% decrease in muscle mass, when compared to being OMNI, (b) this decrease in % muscle mass was attenuated in VEG, despite this group having the lowest intake of dietary protein, and that c) the prevalence of B12 and iron supplementation were strikingly different between dietary patterns, we hypothesized that these micronutrient supplementation habits could underlie, at least in part, the observed differences in % muscle mass.

In fact, in our sample, blood levels of B12 vitamin correlated negatively with blood homocysteine (*r_s_* = −0.386, *p* < 0.001) and positively with % muscle mass (*r_s_* = 0.136, *p* = 0.005) (Table 6).

On the other hand, oral iron supplementation has been suggested to induce adverse effects due to the presence of unabsorbed iron throughout the intestine, which may cause oxidative stress, lipid peroxidation, and mitochondrial damage while affecting the intestinal microbiota and promoting inflammation [43], which is negatively associated with muscle mass, as shown by a recent meta-analysis [44].

In agreement with this, in our sample as displayed in Table 5, participants using iron supplements presented higher C-reactive protein (CRP) (*p* = 0.014), and they had lower % MM (*p* = 0.003).

## 4. Discussion

To the best of our knowledge, this is the first study characterizing anthropometric and body composition differences between strict vegetarians, lacto-ovo-vegetarians, and omnivorous adults living in Portugal and exploring the nutritional and health parameters underlying the observed differences. Published literature presents comparisons of vegetarians and omnivores in other countries [26,40,45,46,47,48,49,50], yet food culture and availability and dietary choices significantly differ across countries, requiring country-specific actions.

We observed that a relevant proportion of participants incorrectly identified their own dietary pattern (8% of the total sample). Despite all the OMNI individuals having correctly self-reported their dietary pattern, some of the self-reported LOV and VEG individuals did not, as assessed by their answers to the FFQ. This suggests some degree of food illiteracy regarding the definition of vegetarian diets, which is surprising given the high degree of education observed among study participants, with the large majority having university studies [51]. Considering the known association between food illiteracy and worse [52] or unhealthier food choices [53], the herein observed lack of knowledge on vegetarian diets could contribute to failure in meeting nutritional needs, leading to health risks.

Nevertheless, in our study, the LOV and VEG groups sought more nutritional counselling, and they had a higher frequency of isolated micronutrient supplement intake than the OMNI group. In fact, and especially for more strict dietary patterns, the long-term success of a healthy diet and the guarantee of the nutritional needs can increase with the follow-up from nutrition specialists [54]. Also, a significantly higher proportion of vegetarians compared to omnivorous individuals taking micronutrient supplements was also noted in previous studies [55,56,57,58], especially with regards to vitamin B12. This suggests that vegetarians may be aware of the possible negative health impacts of their stricter diet, thus adopting behaviors to tackle this potential problem. Importantly, our study indicates a higher prevalence of B12 supplementation among vegans (93%), when compared with other studies [55,57,58] showing a prevalence between 20% and 52%.

Surprisingly, in our study, the prevalence of iron supplementation was the highest among LOV participants. This was not the case in a recent study of Norwegian participants, which shows a higher prevalence of iron supplementation among vegans (11%) when compared to lacto-ovo-vegetarians (4%) [59].

In our study, being LOV was associated with having a higher fat mass and a lower % muscle mass when compared to being OMNI. In VEG, these differences followed the same direction, but they were of smaller magnitude and with no statistical significance. Importantly, the observed association between LOV diet and fat mass may be due to the higher intake of total carbohydrate and a trend for higher intake of total fat and total energy, which was less evident or absent in the VEG group. Literature from observational research often shows disparities in results regarding the association between plant-based diets and fat mass [12], and evidence from experimental studies in healthy populations is scarce [12]. A comparative study between vegetarian and omnivorous adults found no difference on % fat mass between vegetarians and omnivores, yet the sample size (n = 44) was much smaller than ours, and consumption of energy-dense foods was not different between groups [40]. Another study on adult Koreans found that vegetarians had a significantly lower body fat percentage than that of omnivores, which could be justified by a lower total fat intake [60], which was not observed in our study.

Muscle mass was 2.2% lower in LOV compared to OMNI. This result could be partially explained by the lower intake of food protein observed for vegetarian groups when compared to omnivorous individuals. In fact, some studies with a large number of vegetarians have found protein intakes to be lower in this diet group when compared to non-vegetarians [20,48] and low protein intake associates with low muscle mass [61]. However, in our findings, the decrease in % muscle mass was surprisingly more pronounced for LOV and not for VEG, as could be expected by the observed pattern of food protein intake.

In our sample, two lifestyle differences between dietary pattern groups might explain this unexpected observation. First, the higher prevalence of B12 vitamin supplementation by VEG, which was reflected on a better B12 body status, might attenuate the decrease in muscle mass induced by the lower protein intake. In fact, B12 intake has been positively associated with muscle mass in older adults [62] and in adult women with obesity [63]. On the other hand, despite data on associations of B12 vitamin status and muscle mass being scarce in samples of general adult populations, studies on older adults show that vitamin B12 deficiency is associated with decreased muscle mass and a lower skeletal muscle mass index [64] and that in older sarcopenic women, B12 insufficiency negatively impacts physical performance and increases the sarcopenia’s incidence [65].

In our study, B12 status was not only positively correlated with % muscle mass but it was also negatively correlated with homocysteinemia, which has been suggested to explain the association between B12 status and muscle mass. In fact, high levels of blood homocysteine, resulting from low blood levels of B12, are thought to increase oxidative stress, leading to mitochondrial loss and thus impacting skeletal muscle structure and function [66,67].

Additionally, we suggest that the higher prevalence of iron supplementation among LOV could underlie, at least in part, the observed lower % muscle mass in this diet group. In our sample, iron status, as measured by blood ferritin, was lower in vegetarian dietary patterns despite a higher iron supplementation in LOV. So, iron status per se could not explain the putative effect of iron supplementation on muscle mass loss. However, it is known that a high oral iron intake, such as the one resulting from supplementation, may induce adverse effects due to the accumulation of unabsorbed iron throughout the intestine, causing intestinal epithelial cell destruction by iron-induced ROS, which leads to an incomplete intestinal mechanical barrier [68]. This event causes oxidative stress, lipid peroxidation, and mitochondrial damage while affecting the intestinal microbiota and promoting or exacerbating inflammation [43]. Additionally, inflammation is negatively associated with muscle mass, as evidenced in a recent meta-analysis [44]. Curiously in our sample, not only did iron supplementation associate with lower muscle mass and with higher blood levels of C-reactive protein, but also higher C-reactive protein associated with lower muscle mass. So, we suggest that the more pronounced decrease in muscle mass observed in LOV could be explained by an inflammatory status resulting from a putative excessive iron supplementation.

### Strengths and Limitations

The major strength of VeggieNutri is that it is the first study, to our knowledge, to characterize the differences in anthropometry and body composition between the OMNI, LOV, and VEG groups of adults living in Portugal. Importantly, this is also the first study implicating iron supplementation and the associated inflammation as players in the determination of muscle mass in vegetarians. Our data represents the kick-off for new opportunities to study in depth the vegetarian diets as emerging dietary patterns and their associations with health outcomes in the country.

In our study, most sociodemographic and lifestyle characteristics were not significantly different between the three dietary pattern groups, which indicates that our sample groups are not excessively heterogeneous and comparisons can be made with a high degree of certainty.

Additionally, this study benefits from a wide range of anthropometrics, body composition measures, and health variables, namely blood measurements, assessed using standardized procedures, which enhances the internal validity of the study.

As for limitations, our study is based on a convenience sample, which is not representative of the whole population living in Portugal due to two major reasons: first, the vast majority of our participants lived in Northern Portugal, which was expected since the participation in our study was face-to-face in our recruitment center in Porto, North of Portugal; and second, our sample is composed of volunteers with potentially greater concerns about their health and hence being fairly healthy and from a more advantageous socioeconomic position. The relatively low sample sizes, in particular in the vegan group, might have limited to detection of some associations. This reflects the lower prevalence of these diets at the population level and the difficulty in recruiting them to face-to-face assessments.

The use of BIA can be identified as another limitation since this is not considered the gold-standard method for body composition assessment. Nevertheless, BIA has been described as a reliable and valid method, and it was chosen for this study due to its availability, practicality, and fastness of employment [36].

For food intake assessment, we used an FFQ, which tends to overestimate specific food groups, in particular those less frequently consumed [69]. Additionally, the Portuguese FFQ herein used does not assess ingested water, evaluating only water ingestion through food consumption. However, alternative tools such as the 24 h recall could not be used because the data generated by using this alternative method may not represent the long-term dietary habits of participants [69], which was mandatory in this study.

## 5. Perspectives and Future Directions

Our results represent the kick-off to modulate vegetarian habits in Portugal and to empower healthcare professionals and public health entities to act on the best practices of dietary guidance for these specific population groups.

Particularly in Portugal, there is still a general lack of research on the health effects of plant-based diets in healthy adults and also in specific population groups such as pregnant women, children, and older individuals. Additionally, the diet quality of vegetarians in Portugal is still unknown. Despite all this research, which can more easily be conducted in observational studies, future experimental studies employing plant-based diets as interventions in the context of controlled feeding are needed to identify causal relations between plant-based diets and specific metabolic and health outcomes.

The associations between the intake of B12 vitamin and iron supplements and body composition, especially muscle mass status, in general adult populations should be further investigated in order to better understand the impacts of the consumption of over-the-counter nutritional supplements in healthy adults.

## 6. Conclusions

Our study shows that, despite literature pointing out several health benefits of well-planned vegetarian dietary patterns, these diets may not be associated with better health, anthropometric, and body composition profiles. In the current study, plant-based diets were associated with a lower % muscle mass, which could be induced by lower food protein intake. However, the observed lower % muscle mass seemed to be attenuated in vegans, most likely due to B12 supplementation and/or aggravated in lacto-ovo-vegetarians due to an increased inflammatory status associated with a higher prevalence of iron supplementation observed in this sample group. The molecular mechanisms underlying this intricate relationship between micronutrient supplementation and muscle mass deserve to be further explored.

This study may contribute to informe healthcare professionals and public health entities, empowering them to act on the best practices of dietary guidance for vegetarian population groups in Portugal.

## Figures and Tables

**Table 1:** Sociodemographic and lifestyle characteristics of the overall study sample and by dietary pattern.

	Total Sample (n = 425)	OMNI (n = 263)	LOV (n = 98)	VEG (n = 64)	*p*
**Age**					
Mean ± SD, years	36 ± 12	37 ± 12	33 ± 10	32 ± 9	**0.001** ^a^
Min–max	18–63	18–63	18–62	20–52	
**Sex**					
Male	117 (28)	74 (28)	22 (22)	21 (33)	0.331 ^b^
Female	308 (72)	189 (72)	76 (78)	43 (67)	
**Marital status, n (%)**					
Single	256 (61)	146 (56)	69 (71)	41 (64)	**0.028** ^b^
Married or **de facto** union	136 (32)	90 (34)	25 (26)	21 (33)	
Divorced or widowed	31 (7)	26 (10)	3 (3)	2 (3)	
Physical exercise *, n (%)					
No	156 (37)	102 (39)	31 (32)	23 (36)	0.451 ^b^
Yes	269 (63)	161 (61)	67 (68)	41 (64)	
**Physical exercise frequency, n (%)**					
up to 2×/week	99 (37)	66 (41)	22 (33)	11 (27)	0.293 ^b^
3 to 4×/week	106 (39)	62 (39)	28 (42)	16 (39)	
≥5x/week	64 (24)	33 (20)	17 (25)	14 (34)	
**Supplement intake, n (%)**					
No	230 (55)	175 (67)	46 (47)	9 (14)	**0.001** ^b^
Yes	191 (45)	85 (33)	51 (53)	55 (86)	
**Multivitamin**					
No	141 (74)	50 (60)	41 (80)	50 (91)	**0.001** ^b^
Yes	49 (26)	34 (41)	10 (20)	5 (9)	
**B12 vitamin**					
No	95 (50)	70 (83)	21 (41)	4 (7)	**0.001** ^b^
Yes	95 (50)	14 (17)	30 (59)	51 (93)	
**Omega-3**					
No	154 (81)	68 (81)	42 (82)	44 (80)	0.953 ^b^
Yes	36 (19)	16 (19)	9 (18)	11 (20)	
**Iron**					
No	156 (82)	72 (86)	36 (71)	48 (87)	**0.042** ^b^
Yes	34 (18)	12 (14)	15 (29)	7 (13)	
**Selenium**					
No	186 (98)	81 (96)	51 (100)	54 (98)	0.369 ^b^
Yes	4 (2)	3 (4)	0 (0)	1 (2)	
**Calcium**					
No	184 (97)	82 (98)	49 (96)	53 (96)	0.859 ^b^
Yes	6 (3)	2 (2)	2 (4)	2 (4)	
**Zinc**					
No	183 (96)	80 (95)	50 (98)	53 (96)	0.704 ^b^
Yes	7 (4)	4 (5)	1 (2)	2 (4)	
**Magnesium**	190	84	51	55	
No	163 (86)	69 (82)	46 (90)	48 (87)	0.401 ^b^
Yes	27 (14)	15 (18)	5 (9)	7 (13)	
**Protein supplementation**					
No	408 (96)	253 (96)	95 (97)	60 (94)	0.598 ^c^
Yes	17 (4)	10 (4)	3 (3)	4 (6)	
**Nutritional counseling, n (%)**					
No	340 (80)	228 (87)	67 (68)	45 (70)	**0.001** ^b^
Yes	84 (20)	34 (13)	31 (32)	19 (30)	
Doctor (only)	3 (4)	2 (6)	0 (0)	1 (5)	0.476 ^c^
Nutritionist (only)	73 (86)	29 (85)	29 (94)	15 (79)	
Homeopath (only)	3 (4)	0 (0)	1 (3)	2 (11)	
Doctor and Nutritionist	3 (4)	1 (3)	1 (3)	1 (5)	
Other	2 (2)	2 (6)	0 (0)	0 (0)	

Legend: LOV, lacto-ovo-vegetarians; Max, maximum; Min, minimum; OMNI, omnivorous; VEG, vegans. Bold notation in *p* values indicates highlights statistical significance. Total missings between 0% and 14%, * Physical exercise = planned, structured, and regular physical activity aiming to keep or improve physical performance, ^a^ ANOVA, ^b^ Chi-square test, ^c^ Fisher’s exact test.

**Table 2:** Anthropometric and body composition characteristics of the overall study sample and by dietary pattern.

Characteristics	Total Sample (n = 425)	OMNI (n = 263)	LOV (n = 98)	VEG (n = 64)	*p*
**Weight**					
Median (P25; P75), kg	63.5 (56.2; 73.8)	64.0 (56.4; 74.4)	65.4 (57.7; 71.9)	61.0 (53.3; 74.7)	0.384 ^a^
**Height**					
Mean ± SD, cm	166.5 ± 8.9	166.3 ± 8.9	166.7 ± 7.2	167.4 ± 9.7	0.637 ^b^
**BMI**					
Median (P25; P75), kg/m^2^	23.2 (21.0; 25.6)	23.3 (21.4; 25.6)	23.4 (20.9; 25.6)	22.3 (20.3; 25.7)	0.118 ^a^
Underweight	9 (2)	3 (1)	2 (2)	4 (6)	
Normal weight	274 (65)	171 (65)	62 (63)	41 (64)	0.352 ^c^
Overweight	111 (26)	70 (27)	25 (26)	16 (25)	
Obese	31 (7)	19 (7)	9 (9)	3 (5)	
**Waist circumference (WC)**					
Median (P25; P75), cm	82.0 (74.6; 89.4)	83.4 (76.6; 90.3)	80.2 (72.2; 86.8)	79.7 (72.8; 88.6)	**0.010** ^a^
**Fat mass (FM) ^#^**					
Median (P25; P75), %	25 (20; 30)	24.5 (19.6; 29.8)	26.3 (20.2; 31.2)	24.3 (17.9; 28.2)	0.082 ^a^
**Visceral Fat (VF)**					
Median (P25; P75)	3.5 (2.0; 6.5)	4.0 (2.0; 6.5)	3.5 (1.5; 5.0)	3.0 (1.5; 5.0)	**0.033** ^a^
**Bone mass (BM)**					
Median (P25; P75), kg	2.4 (2.2; 2.8)	2.4 (2.2; 2.9)	2.4 (2.2; 2.7)	2.3 (2.1; 2.9)	0.471 ^a^
**Muscle mass (MM)**					
Median (P25; P75), kg	44.1 (40.3; 52.8)	44.6 (40.4; 54.4)	44.1 (40.9; 50.1)	43.0 (39.1; 54.5)	0.534 ^a^
**Muscle mass adjusted for body weight (%MM)**					
Mean ± SD, %	71.3 ± 7.4	71.4 ± 7.2	70.1 ± 7.9	72.6 ± 7.1	0.101 ^b^
**Total body water (TBW) ^#^**					
Median (P25; P75), %	51.7 (47.6; 55.6)	51.3 (47.5; 55.2)	51.1 (47.1; 55.3)	53.6 (51.3; 57.0)	**0.009** ^a^

Legend: LOV, lacto-ovo-vegetarians; OMNI, omnivorous; P25, 25th percentile; P75, 75th percentile; SD, standard deviation; VEG, vegans. Bold notation in *p* values highlights statistical significance. Missings between 0% and 0.5% in all variables except for HGS, whose missings were from 37% to 64%. ^a^ Kruskal–Wallis test, ^b^ ANOVA test, and ^c^ Fisher’s exact test. ^#^ FM and TBW given in % of total body weight. Cut-off points for BMI classes as defined by the WHO BMI classification [37].

**Table 3:** Multivariable linear regression models for the association between dietary patterns and anthropometry, body composition, and health measurements.

	OMNI	LOV			VEG		
		Beta ^a^	95% CI	*p*	Beta ^a^	95% CI	*p*
Weight, kg	Ref	1.615	(−2.516; 5.747)	0.444	−0.466	(−5.337; 4.404)	0.851
Height, cm	Ref	2.010	(−0.0333; 4.354)	0.093	0.176	(−2.597; 2.950)	0.901
BMI, kg/m^2^	Ref	−0.142	(−1.152; 1.435)	0.830	−0.342	(−1.867; 1.183)	0.660
Waist circumference, cm	Ref	−2.006	(−5.481; 1.468)	0.258	−0.971	(−5.083; 3.142)	0.644
Visceral Fat	Ref	−0.058	(−0.800; 0.685)	0.879	0.067	(−0.809; 0.942)	0.881
Fat Mass ^#^, %	Ref	4.832	(1.774; 7.891)	**0.002**	1.549	(−2.056; 5.155)	0.400
Bone Mass, kg	Ref	−0.011	(−0.119; 0.097)	0.843	−0.065	(−0.192; 0.063)	0.320
Muscle Mass, kg	Ref	−0.408	(−2.555; 1.740)	0.710	−1.030	(−3.562; 1.501)	0.425
% Muscle Mass, %	Ref	−2.232	(−4.396; −0.068)	**0.043**	−0.702	(−3.253; 1.848)	0.589
Hand Grip strength, kgf	Ref	1.063	(−1.759; 3.885)	0.460	−0.216	(−3.627; 3.188)	0.900
Systolic blood pressure, mmHg	Ref	−1.258	(−5.757; 3.240)	0.584	−1.801	(−7.081; 3.479)	0.504
Diastolic blood pressure, mmHg	Ref	1.255	(−1.696; 4.205)	0.405	−0.393	(−3.855; 3.070)	0.824
Total body water ^#^, %	Ref	−0.987	(−2.842; 0.867)	0.297	1.125	(−1.061; 3.311)	0.313

Legend: CI, confidence intervals; LOV, lacto-ovo-vegetarians; OMNI, omnivorous; Ref, reference; VEG, vegans. Bold notation in *p* values highlights statistical significance. ^a^ Model adjusted for sex, age, marital status, physical exercise, multivitamin supplement intake, B12 vitamin supplement intake, iron supplement intake, and nutritional counseling. ^#^ FM and TBW given in % of total body weight.

**Table 4:** Dietary intake and blood biomarkers overall and by dietary pattern.

	Total (n = 425)	OMNI (n = 263)	LOV (n = 98)	VEG (n = 64)	*p*
**Nutrient intake**					
Total water from foods *					
Median (P25; P75), mL/d	1410 (1089; 1851)	1377 (1061; 1836)	1527 (1133; 1835)	1390 (1095; 1945)	0.403 ^a^
**Total energy intake**					
Median (P25; P75), Kcal/d	2184.2 (1716.3; 2737.2)	2119.4 (1668.2; 2583.1)	2344.1 (1901.4; 2856.2)	2168.8 (1711.8; 2900.0)	0.125 ^a^
**Total carbohydrate intake**					
Median (P25; P75), g/d	249.1 (195.5; 326.0)	231.5 (178.4; 287.9)	283.9 (227.3; 342.6)	263.0 (222.0; 348.3)	**0.001** ^a^
**Total fat intake**					
Median (P25; P75), g/d	87.6 (67.3; 116.8)	85.7 (67.5; 113.8)	94.4 (66.9; 129.0)	81.9 (66.6; 110.1)	0.338 ^a^
**Total protein intake**					
Median (P25; P75), g/d	95.4 (72.7; 122.1)	98.6 (79.5; 123.1)	90.4 (65.9; 121.0)	87.6 (59.8; 118.5)	**0.020 ^a^**
**Protein intake adequacy ^#^**					
below adequacy, n (%)	5 (1.2)	0 (0.0)	2 (2.0)	3 (4.7)	
within adequacy, n (%)	420 (98.8)	263 (100.0)	96 (98.0)	61 (95.3)	**0.004 ^b^**
above adequacy, n (%)	0 (0.0)	0 (0.0)	0 (0.0)	0 (0.0)	
**Total iron intake**					
Median (P25; P75), mg/d	18.8 (13.8; 25.7)	16.2 (12.7; 21.7)	22.9 (18.1; 29.4)	23.2 (17.8; 32.7)	**0.001 ^a^**
**Total B12 vitamin intake**					
Median (P25; P75), µg/d	4.7 (1.3; 9.2)	8.0 (5.2; 11.5)	1.4 (0.8; 1.8)	0.5 (0.3; 0.9)	**0.001 ^a^**
**Blood biomarkers**					
**Blood level of iron**					
Median (P25; P75), µg/dL	98 (73; 121)	98 (74; 118)	92 (69; 118)	107 (76; 135)	0.117 **^a^**
**Blood level of ferritin**					
Median (P25; P75), ng/mL	52 (27; 100)	61 (31; 121)	42 (25; 61)	40 (22; 72)	**0.001 ^a^**
**Blood level of B12 vitamin**					
Median (P25; P75), pg/mL	373 (287; 496)	389 (316; 532)	310 (234; 399)	412 (281; 536)	**0.001 ^a^**

Legend: LOV, lacto-ovo-vegetarians; OMNI, omnivorous; P25, 25th percentile; P75, 75th percentile. VEG, vegans. Bold notation in *p* values highlights statistical significance. ^a^ Kruskal–Wallis test; ^b^ Fisher’s exact test * Percentage of total water ingestion through food consumption; ^#^ protein intake adequacy categories based on the IOM *Recommended Dietary Allowances and Adequate Intakes, Total Water, and Macronutrients* [32]. Missings were 0% on FFQ data and 0.2% on all variables from the blood sample analysis.

**Table 5:** Blood iron, ferritin, C-reactive protein (CRP), and % muscle mass (MM) according to iron oral supplementation in the overall study sample.

	Iron Supplement		
	Non-Users	Users	*p*
**Blood level of iron, n**	156	33	
Median (P25; P75), µg/dL	99.0 (77.5; 126.0)	87.0 (71.0; 115.0)	0.215 ^a^
**Blood level of ferritin, n**	156	33	
Median (P25; P75), ng/mL	49.2 (30.6; 87.2)	42.4 (26.8; 58.6)	0.133 ^a^
**CRP, n**	156	33	
Median (P25; P75), mg/L	1.0 (0.5; 2.1)	2.1 (0.8; 6.7)	**0.014** ^a^
**% MM, n**	155	34	
mean ± SD, %	72.6 ± 7.1	68.6 ± 7.0	**0.003** ^b^

Legend: CRP, C-Reactive Protein; % MM, percentage muscle mass; P25, 25th percentile; P75, 75th percentile; SD, standard deviation. Bold notation in *p* values highlights statistical significance. ^a^ Mann–Whitney, ^b^ ANOVA.

**Table 6:** Correlations of B12 blood levels with homocysteinemia or % muscle mass in the overall study sample.

	Blood Level of Homocysteine (μmol/mL)			% Muscle Mass		
	n	*r_s_*	*p*	n	*r_s_*	*p*
**Blood level of B12 vitamin (pg/mL)**	418	−0.386	**<0.001** ^a^	423	0.136	**0.005** ^a^

Bold notation in *p* values highlights statistical significance. ^a^ Spearman correlation test.

## Data Availability

Data generated or analyzed during this study are included in this published article or in the respective Supplementary Materials.

## Supplementary Materials

The following supporting information can be downloaded at: https://www.mdpi.com/article/10.3390/nu16193322/s1. Table S1: Variations between self-reported diet groups (OMNI, VEG, LOV) and real diet groups, as confirmed by the FFQ; Table S2: Analysis on subgroups based on the duration of diet adherence to assess its impact on body composition and anthropometric parameters; Table S3: Analysis on subgroups based on the duration of diet adherence to assess its impact on biochemical parameters; Table S4: Crude linear regression models for the association between dietary patterns and body composition, anthropometric, and health measurements; Table S5: Sex-specific reference levels for blood ferritin and blood B12 vitamin set by the Clinical Pathology Department of CHUSJoão, Porto, Portugal that were used as cut-offs of adequacy.
