# Supplementary material for: The Relevance of Plant-Based Diets and Micronutrient Supplementation for Body Composition: Data from the VeggieNutri Cross-Sectional Study
Source: Nutrients. 2024 Sep 30;16(19):3322. doi: 10.3390/nu16193322 (PMC11478620; doi:10.3390/nu16193322)
Supplement: Supplementary file 1 [file nutrients-16-03322-s001.zip › nutrients-3155970-supplementary.pdf]

nutrients

MDPI

*Supplementary Materials*

# The Relevance of Plant-Based Diets and Micronutrient Supplementation for Body Composition: Data from the VeggieNutri Cross-Sectional Study

**Table S1.** Variations between self-reported diet groups (OMNI, VEG, LOV) and real diet groups, as confirmed by the FFQ.

| | Self-reported diet | Real diet | Total variation | Group variation |
|---|---|---|---|---|
| **Dietary pattern, n** | 425 | 425 | | |
| **OMNI, n (%)** | 247 (58) | 263 (62) | +16 (+4%) | + 14 from LOV |
| | | | | + 2 from VEG |
| **LOV, n (%)** | 106 (25) | 98 (23) | -8 (-2%) | - 1 to VEG |
| | | | | - 14 to OMNI |
| | | | | + 7 from VEG |
| **VEG, n (%)** | 72 (17) | 64 (15) | -8 (-2%) | - 2 to OMNI |
| | | | | - 7 to LOV |
| | | | | + 1 from LOV |

Legend: LOV, lacto-ovovegetarians; OMNI, omnivorous; VEG, vegans.

**Table S2.** Analysis on subgroups based on the duration of diet adherence to assess its association with anthropometric parameters, body composition and age.

| | LOV | | | VEG | | |
|---|---|---|---|---|---|---|
| | **diet duration ≤ 5 years** | **diet duration > 5 years** | *p* | **diet duration ≤ 5 years** | **diet duration > 5 years** | *p* |
| **Weight** | | | | | | |
| Median (P25; P75), kg | 63 (57.7; 73.9) | 65.5 (57.5; 61.4) | 0.871 [a] | 62.2 (52.7; 75.1) | 59.9 (54.6; 78.9) | 0.811 [a] |
| **Height** | | | | | | |
| Mean ± SD, cm | 166.5 ± 7.6 | 167.6 ± 6.3 | 0.768 [b] | 167.8 ± 9.7 | 167.7 ± 8.9 | 0.699 [b] |
| **BMI** | | | | | | |
| Median (P25; P75), kg/m$^2$ | 23.4 (21.2; 25;5) | 23.3 (20.5; 25.6) | 0.856 [a] | 22.9 (20.4; 25.7) | 21.9 (20.6; 26.5) | 1.000 [a] |
| **Waist circumference** | | | | | | |
| Median (P25; P75), cm | 79.3 (71.8; 86.0) | 80.7 (74.6; 86.8) | 0.236 [a] | 80.1 (72.5; 88.4) | 79.7 (75.1; 89.0) | 0.794 [a] |
| **Fat mass** | | | | | | |
| Median (P25; P75), % | 26.7 (19.9; 30.7) | 25.8 (21.6; 33.0) | 0.670 [a] | 24.3 (20.1; 28.9) | 25.1 (17.8; 28.0) | 0.741 [a] |
| **Visceral Fat** | | | | | | |
| Median (P25; P75) | 3.0 (1.5; 4.5) | 4.0 (3.0; 5.5) | **0.039** [a] | 3.0 (1.5; 5.0) | 3.3 (2.0; 5.3) | 0.620 [a] |
| **Bone mass** | | | | | | |
| Median (P25; P75), kg | 2.4 (2.2; 2.7) | 2.3 (2.2; 2.6) | 0.833 [a] | 2.3 (2.1; 3.0) | 2.3 (2.1; 2.9) | 0.969 [a] |
| **Muscle mass** | | | | | | |
| Median (P25; P75), kg | 44.3 (41.2; 50.1) | 43.8 (40.9; 48.7) | 0.837 [a] | 43.1 (38.6; 57.6) | 42.8 (39.7; 53.3) | 0.829 [a] |
| **Muscle mass adjusted for body weight** | | | | | | |
| Mean ± SD, % | 70.8 ± 8.0 | 69.8 ± 7.1 | 0.135 [b] | 72.5 ± 6.9 | 71.7 ± 7.5 | 0.740 [b] |
| **Total body water** | | | | | | |
| Median (P25; P75), % | 51.5 (47.3; 56.1) | 51.2 (47.1; 54.7) | 0.564 [a] | 54.0 (51; 55.8) | 51.9 (51.3; 57.5) | 0.569 [a] |
| **Age** | | | | | | |
| Mean ± SD, years | 31.2 ± 9.3 | 35.9 ± 10.3 | **0.026** [a] | 31.7 ± 9.0 | 32.5 ± 7.4 | 0.498 [a] |

Legend: LOV, lacto-ovovegetarians; VEG, vegans. [a] Mann-Whitney test; [b] ANOVA.

**Table S3.** Analysis on subgroups based on the duration of diet adherence to assess its impact on biochemical parameters.

| | LOV | | | VEG | | |
|---|---|---|---|---|---|---|
| | **diet duration ≤ 5 years** | **diet duration > 5 years** | ***p*** | **diet duration ≤ 5 years** | **diet duration > 5 years** | ***p*** |
| **B12 vitamin** | | | | | | |
| Median (P25; P75), (pg/mL) | 309 (230; 407) | 320 (234; 391) | 0.729 [a] | 400 (278; 507) | 407 (298; 568) | 0.883 [a] |
| **Iron** | | | | | | |
| Median (P25; P75), (μg/mL) | 95 (65; 125) | 93 (75; 115) | 0.997 [a] | 108 (77; 131) | 106 (77; 153) | 0.666 [a] |
| **C-Reactive protein** | | | | | | |
| Median (P25; P75), (mg/mL) | 2.2 (0.8; 3.7) | 1.1 (0.6; 3.2) | 0.260 [a] | 0.7 (0.4; 2.8) | 0.7 (0.4; 1.1) | 0.558 [a] |
| **Homocysteine** | | | | | | |
| Median (P25; P75), (μmol/mL) | 9.0 (6.7; 10.4) | 9.5 (8.2, 11.3) | 0.167 [a] | 8.5 (7.3; 21.1) | 8 (7; 9) | 0.230 [a] |

Legend: LOV, lacto-ovovegetarians; VEG, vegans. [a] Mann-Whitney test.

**Table S4.** Crude linear regression models for the association between dietary patterns and body composition, anthropometric, health measurements.

| | OMNI | LOV | | | VEG | | |
|---|---|---|---|---|---|---|---|
| | | Beta | 95% CI | p | Beta | 95% CI | p |
| Weight, kg | Ref | -0.805 | (-3.834; 2.223) | 0.602 | -1.652 | (-5.206; 1.902) | 0.362 |
| Height, cm | Ref | 0.406 | (-1.608; 2.419) | 0.693 | 1.209 | (-1.238; 3.504) | 0.349 |
| BMI, kg/m$^2$ | Ref | 0.289 | -1.178; 0.599) | 0.523 | -1.077 | (23.626; 24.548) | **0.043** |
| Waist circumference, cm | Ref | -0,778 | (-1.492; -0.063) | **0.033** | -1.042 | (-1.880; -0.203) | **0.015** |
| Visceral Fat | Ref | -3,48 | (-6.103; -0.850) | **0.009** | -2.989 | (-6.082; 0.104) | **0.058** |
| Fat Mass, % | Ref | 2.276 | (0.369; 4.183) | **0.019** | -1.146 | (-3.384; 1.091) | 0.315 |
| Bone Mass, kg | Ref | -0.082 | (-0.190; 0.026) | 0.137 | -0.044 | (-0.171; 0.083) | 0.497 |
| Muscle Mass, kg | Ref | -1.670 | (-3.853; 0.512) | 0.134 | -0.521 | (-3.082; 2.040) | 0.690 |
| % Muscle Mass, % | Ref | -1.314 | (-3.018; 0.390) | 0.131 | 1.171 | (-0.829; 3.170) | 0.251 |
| Total Body Water, % | Ref | 0.016 | (-1.346; 1.379) | 0.981 | 2.438 | (0.839; 4.037) | **0.003** |
| Handgrip strenght, kgf | Ref | 0.346 | (-2.197; 2.888) | 0.790 | 1.643 | (-1.545; 4.831) | 0.313 |
| Systolic blood pressure, mmHg | Ref | -2.504 | (-5.787; 0.780) | 0.135 | -1.465 | (-5.303; 2.373) | 0.454 |
| Diastolic blood pressure, mmHg | Ref | -0.857 | (-2.988; 1.274) | 0.431 | -1.583 | (0.908; 1.552) | 0.213 |

Legend: BMI, body mass index; CI, confidence interval; LOV, lacto-ovovegetarians; OMNI, omnivorous; Ref, reference; VEG, vegans.

**Table S5.** Sex-specific reference levels for blood ferritin and blood B12 vitamin set by the Clinical Pathology Department of CHUSJoão, Porto, Portugal that were used as cut-offs of adequacy.

| **Ferritin adequacy** | **MALE** |
|---|---|
| above adequacy | > 200 ng/mL |
| within adequacy | 15 – 200 ng/mL |
| below adequacy | < 15 ng/mL |
| **Ferritin adequacy** | **FEMALE** |
| above adequacy | > 150 ng/mL |
| within adequacy | 15 – 150 ng/mL |
| below adequacy | < 15 ng/mL |
| **B12 vitamin adequacy** | **MALE/FEMALE** |
| above adequacy | > 883 pg/mL |
| within adequacy | 187– 883 pg/mL |
| below adequacy | < 187 pg/mL |
